# Synthesis, Characterization, and Cytotoxicity of the First Oxaliplatin Pt(IV) Derivative Having a TSPO Ligand in the Axial Position

**DOI:** 10.3390/ijms17071010

**Published:** 2016-06-25

**Authors:** Salvatore Savino, Nunzio Denora, Rosa Maria Iacobazzi, Letizia Porcelli, Amalia Azzariti, Giovanni Natile, Nicola Margiotta

**Affiliations:** 1 Dipartimento di Chimica, Università degli Studi di Bari Aldo Moro, via E. Orabona 4, 70125 Bari, Italy; salvatoresavino.s@libero.it (S.S.); giovanni.natile@uniba.it (G.N.); 2 Dipartimento di Farmacia-Scienze del Farmaco, Università degli Studi di Bari Aldo Moro, via E. Orabona 4, 70125 Bari, Italy; nunzio.denora@uniba.it (N.D.); rosamaria.iacobazzi@libero.it (R.M.I.); 3 Istituto Tumori IRCCS Giovanni Paolo II, viale O. Flacco 65, 70124 Bari, Italy; porcelli.letizia@gmail.com (L.P.); a.azzariti@oncologico.bari.it (A.A.)

**Keywords:** oxaliplatin, antitumor drugs, platinum(IV), translocator protein 18 kDa, TSPO, colorectal cancer

## Abstract

The first Pt(IV) derivative of oxaliplatin carrying a ligand for TSPO (the 18-kDa mitochondrial translocator protein) has been developed. The expression of the translocator protein in the brain and liver of healthy humans is usually low, oppositely to steroid-synthesizing and rapidly proliferating tissues, where TSPO is much more abundant. The novel Pt(IV) complex, *cis*,*trans*,*cis*-[Pt(ethanedioato)Cl{2-(2-(4-(6,8-dichloro-3-(2-(dipropylamino)-2-oxoethyl)imidazo[1,2-*a*]pyridin-2-yl)phenoxy)acetate)-ethanolato}(1*R*,2*R*-DACH)] (DACH = diaminocyclohexane), has been fully characterized by spectroscopic and spectrometric techniques and tested in vitro against human MCF7 breast carcinoma, U87 glioblastoma, and LoVo colon adenocarcinoma cell lines. In addition, affinity for TSPO (IC_50_ = 18.64 nM), cellular uptake (ca. 2 times greater than that of oxaliplatin in LoVo cancer cells, after 24 h treatment), and perturbation of cell cycle progression were investigated. Although the new compound was less active than oxaliplatin and did not exploit a synergistic proapoptotic effect due to the presence of the TSPO ligand, it appears to be promising in a receptor-mediated drug targeting context towards TSPO-overexpressing tumors, in particular colorectal cancer (IC_50_ = 2.31 μM after 72 h treatment).

## 1. Introduction

**Figure 1:**
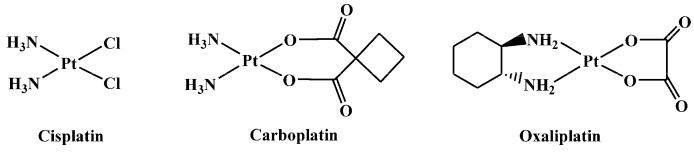
Pt(II) complexes worldwide clinically used.

The discovery of the antitumor activity of cisplatin, *cis*-diamminedichloridoplatinum(II) (Figure 1) [1,2], was a corner stone that triggered the interest in the development of platinum(II)/(IV) complexes in oncology. However, only a few of these complexes—carboplatin and oxaliplatin (Figure 1)—have been approved worldwide for the use in the clinics [3,4,5].

The second generation platinum drug carboplatin, *cis*-diammine(1,1-cyclobutanedicarboxylato)platinum(II), contains a more stable leaving group (1,1-cyclobutanedicarboxylato) with respect to the chlorides present in cisplatin. This modification was introduced to lower the toxicity without affecting the spectrum of antitumor activity. Effectively, carboplatin is less neuro- and nephro-toxic and can be administered at higher doses than cisplatin [6].

The third generation platinum drug oxaliplatin*—trans*-1*R*,2*R*-diaminocyclohexane(oxalato)platinum(II)—contains the diaminocyclohexane (1*R*,2*R*-DACH) chelating ligand and received worldwide approval for the treatment of colorectal cancer [7]. Although oxaliplatin produces the same type of lesions on DNA as cisplatin, its spectrum of activity is different from that of the first-generation drug and also the occurrence of resistance to oxaliplatin is different from that of cisplatin and carboplatin. The mechanism of action responsible for the different activity of oxaliplatin, with respect to cisplatin, has not yet been completely understood. However, it has been demonstrated that the non-leaving diamine has a fundamental role in the recognition of {(DACH)Pt}-DNA adducts by DNA-repair proteins. These adducts, in turn, could contribute to the absence of cross-resistance with the other two Pt-drugs cisplatin and carboplatin [8]. Neurotoxicity is the major dose-limiting feature associated with the use of oxaliplatin [9].

Due to their scarce water solubility, platinum(II) complexes are administered by intravenous infusion in the clinics, with low compliance in treated patients [10]. This drawback can be overcome with platinum(IV)-based drugs which are known to be much more resistant (with respect to Pt(II) complexes) to substitution from biomolecules and are hence suitable for oral administration [11,12]. Pt(IV) compounds are generally considered prodrugs since they must be activated by intracellular reduction to their Pt(II) counterparts, therefore the structure, substituents and reduction potential of Pt(IV) complexes are strictly correlated to their antitumor activity. Hambley and colleagues showed that in a series of ethylenediamine-based Pt(IV) complexes the cathodic potential for the reduction of Pt(IV) to Pt(II) depends upon the nature of the axial ligands and decreases in the order Cl > OCOR > OH [13].

Axial ligands also represent a possible way to link biovectors with the ability to direct the complex toward a tumor. Overall activity would not be altered since these ligands are released upon reduction with generation of the active Pt(II) metabolite [14].

The peripheral-type benzodiazepine receptor (PBR) has been recently re-named 18-kDa mitochondrial translocator protein (TSPO) and has been used as target for several potential drugs with therapeutic and imaging uses [15,16,17,18].

The expression of TSPO in the brain and liver of healthy humans is usually low. On the contrary, in steroid-synthesizing and rapidly proliferating tissues, TSPO is more abundant. In several pathologies such as brain, breast, colon, prostate, and ovarian cancers and in astrocytomas and hepatocellular and endometrial carcinomas, an overexpression of the TSPO has been found [19,20]. Neurodegenerative diseases such as Alzheimer, Parkinson, Huntington, and multiple sclerosis, that are generally associated with inflammatory processes, also show high levels of TSPO expression in activated microglial cells [21].

It appears clear why TSPO gained much attraction for its use as an intracellular target for imaging of pathologic tissues overexpressing this protein and also for selectively targeting the functions associated with mitochondrial activity [22,23,24,25]. Several classes of ligands having high affinity for TSPO have been developed for diagnostic or therapeutic uses and, in some cases, for both (theranostic agents) [21]. Some of us have exploited the 2-phenyl-imidazo[1,2-*a*]pyridine-*N*,*N*-dipropylacetamide scaffold specific of the anxyolitic drug alpidem [22,23,24] and, as an example, the PET imaging ^18^F-labeled agent 2-(2-(4-(2-[^18^F]fluoroethoxy)phenyl)-6,8-dichloroimidazo[1,2-*a*]pyridine-3-yl)-*N*,*N*-dipropylacetamide was designed as a biomarker for the diagnosis of neuroinflammation, neurodegeneration, and tumor progression [26].

**Figure 2:**
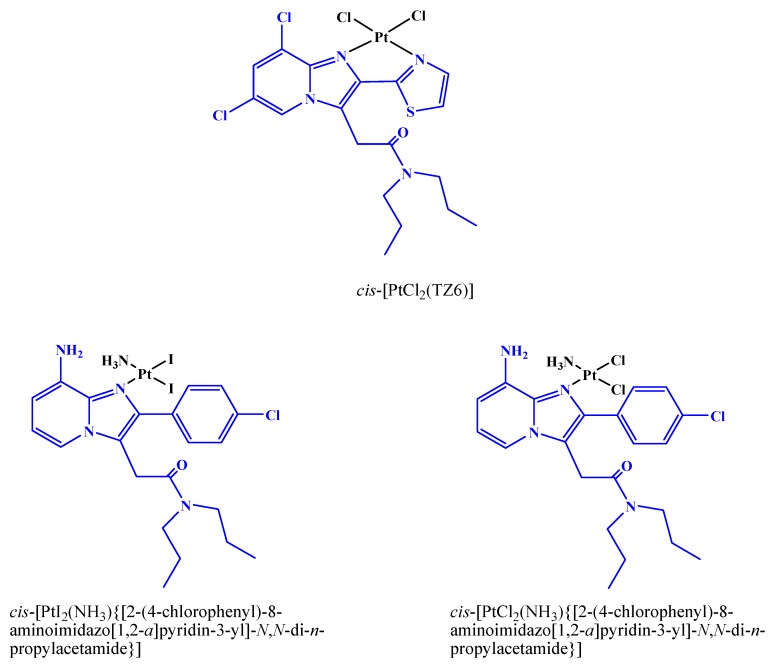
Pt(II) complexes with TSPO ligands (highlighted with a blue color).

Pursuing our interest for tissue-specific targeting of metal complexes, in recent papers we have synthesized platinum(II) compounds with ligands specific for TSPO, such as 2-[6,8-dichloro-2-(1,3-thiazol-2-yl)*H*-imidazo-[1,2-*a*]pyridin-3-yl]-*N*,*N*-dipropylacetamide (TZ6). The resulting cisplatin-like compound, *cis*-[PtCl_2_(TZ6)] (Figure 2), has been shown to possess affinity and selectivity for the TSPO comparable to those of TZ6. In solvents with low dielectric constants, we also observed the formation of a dimeric aggregate formed through non-covalent intermolecular interactions of the planar aromatic cycles of the ligands. This finding further supports the potential intercalating ability of *cis-*[PtCl_2_(TZ6)] toward DNA [27]. The poor aqueous solubility of *cis-*[PtCl_2_(TZ6)], which is typical of platinum(II) complexes with bidentate aromatic ligands [28], compelled us to pursue the synthesis of Pt compounds with TSPO ligands endowed with enhanced water solubility. To this end, in a subsequent work we synthesized two new Pt compounds structurally analogous to picoplatin and differing for the anionic ligands, *cis*-[PtI_2_(NH_3_){[2-(4-chlorophenyl)-8-aminoimidazo[1,2-*a*]pyridin-3-yl]-*N*,*N*-di-*n*-propylacetamide}] and *cis*-[PtCl_2_(NH_3_){[2-(4-chlorophenyl)-8-aminoimidazo[1,2-*a*]pyridin-3-yl]-*N*,*N*-di-*n*-propylacetamide}] (Figure 2) [29]. Both complexes contained the imidazopyridinic ligand [2-(4-chlorophenyl)-8-aminoimidazo-[1,2-*a*]pyridin-3-yl]-*N*,*N*-di-*n*-propylacetamide and were endowed with high affinity and selectivity for TSPO [30].

In addition, the two compounds were massively accumulated in glioma cells (10- to 100-fold enhanced accumulation) and were capable of inducing apoptosis similar to cisplatin [30].

In this work we have developed the first Pt(IV) complex containing a TSPO ligand in axial position (the two axial positions are those perpendicular to the square-planar coordination plane of the platinum(II) precursor).

**Figure 3:**
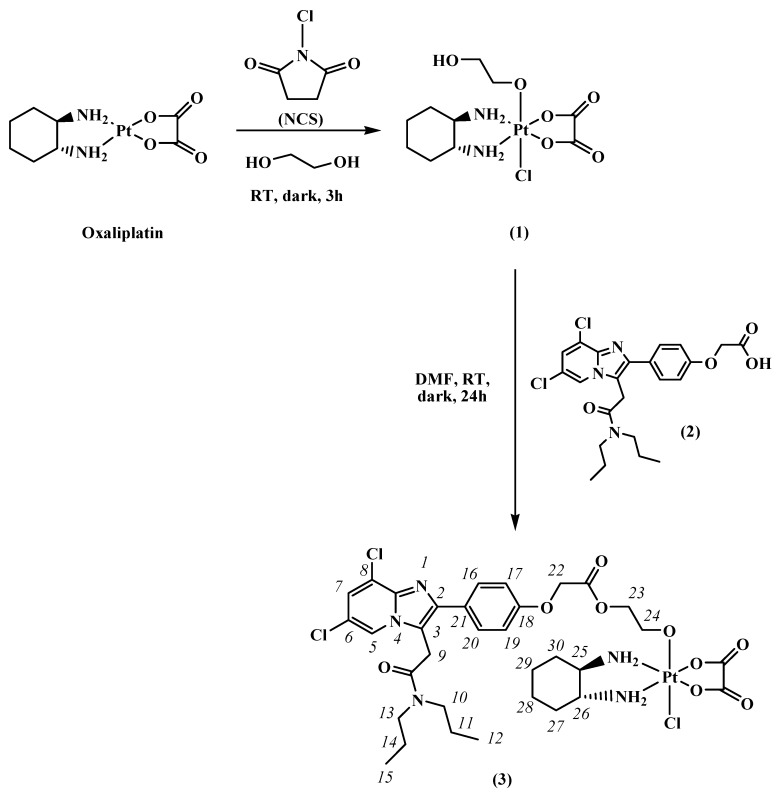
Synthesis of the novel Pt(IV) derivative of oxaliplatin with TSPO ligand in axial position (**3**) and numbering of protons. RT = room temperature.

The design of a Pt(IV) complex with a TSPO ligand in the axial position was motivated by the reasons mentioned earlier in this paragraph: the targeting of the tumor site and the potential synergistic proapoptotic effect caused by the TSPO ligand released by intracellular reduction of the Pt(IV) conjugate. We selected the oxaliplatin derivative *cis*,*trans*,*cis*-[Pt(ethanedioato)Cl(2-hydroxyethanolato)(1*R*,2*R*-DACH)] (DACH = diaminocyclohexane) as Pt(IV) precursor (compound **1** in Figure 3) [31]. The choice of this monofunctional Pt(IV) derivative of oxaliplatin was guided by several considerations: (i) it contains a single reactive group (the terminal hydroxyl moiety of 2-hydroxyethanolato), thus preventing the formation of different condensation products (mono- and di-substituted) in the conjugation with biovectors; (ii) the presence of an axial chloride makes the reduction potential less negative (i.e., “easier” reduction from a thermodynamic point of view) and increases the reduction rate [12,32,33]; (iii) the 2-hydroxyethanolato residue should act as a spacer increasing the distance between the conjugated TSPO ligand and the Pt center, allowing an easier interaction with the receptor.

Hence, the Pt(IV) precursor was tethered to a potent TSPO ligand characterized by a 2-phenyl-imidazo[1,2-*a*]pyridine acetamide structure and containing a terminal carboxylic group useful for its further conjugation, 2-(4-(6,8-dichloro-3-(2-(dipropylamino)-2-oxoethyl)imidazo[1,2-*a*]pyridin-2-yl)phenoxy)acetic acid (compound **2** in Figure 3) [34]. In this way we obtained a novel Pt(IV) derivative of oxaliplatin carrying a ligand for TSPO, *cis*,*trans*,*cis*-[Pt(ethanedioato)Cl{2-(2-(4-(6,8-dichloro-3-(2-(dipropylamino)-2-oxoethyl)imidazo[1,2-*a*]pyridin-2-yl)phenoxy)acetate)-ethanolato}(1*R*,2*R*-DACH)] (compound **3** in Figure 3).

The novel Pt(IV) complex has been fully characterized by spectroscopic and spectrometric techniques and tested in vitro against human MCF7 breast carcinoma, human U87 glioblastoma, and human LoVo colon adenocarcinoma cell lines. In addition, the affinity of **3** for TSPO was assessed by receptor binding assays, measuring its ability to displace [^3^H]-1-(2-chlorophenyl)-*N*-methyl-*N*-(1-methylpropyl)-3-isoquinolinecarboxamide ([^3^H]-PK 11195). Cellular uptake experiments were performed in order to correlate the cytotoxicity of **3** with its cellular uptake ability. Finally, cell cycle analysis was performed to evaluate the capability of **3** to perturb the LoVo cells cycle progression.

## 2. Results and Discussion

### 2.1. Synthesis and Characterization of **3**

Although platinum(IV) complexes were identified by Rosenberg as having anticancer activity at the same time of the discovery of that of cisplatin [1], their clinical efficacy has been tested only recently. The properties of Pt(IV) complexes are substantially different from those of Pt(II) species since six-coordinate octahedral platinum(IV) complexes have a saturated coordination sphere that renders them less prone to substitution reactions than platinum(II) complexes. In addition, the possibility to coordinate two additional ligands in the axial positions enables tuning of the chemical properties and conjugation of cancer-targeting ligands. The preliminar intracellular reduction to Pt(II), with simultaneous release of the two axial ligands, is necessary for Pt(IV) complexes to exert their antitumor activity. Thus, Pt(IV) complexes are generally defined as prodrugs of platinum-based drugs. In addition, the intracellular reduction of Pt(IV) complexes releases the axial ligands that can be themselves biologically active. The Pt(IV) precursor is indeed a dual-threating pharmaceutical agent, which combines two biologically active components into a single molecule [12,14]. In specifically designed Pt(IV) complexes, the Pt-core and the released axial ligands may have different intracellular targets; moreover, the axial ligands may exhibit a targeting property towards substrates that are specifically overexpressed in tumor cells. This is the rationale that prompted us to prepare a Pt(IV) complex with a TSPO ligand in the axial position.

Most of the Pt(IV) complexes present in the literature are prepared via oxidation of predesigned Pt(II) compounds with chlorine [35,36,37] or hydrogen peroxide [38], leading to Pt(IV) complexes with symmetric axial ligands. However, more recently, unsymmetric Pt(IV) complexes have also been synthesized [39,40].

For the preparation of the unsymmetric Pt(IV) derivative of oxaliplatin with just one TSPO ligand in axial position, we first treated oxaliplatin (Figure 3) with *N*-chlorosuccinimide (NCS)—a source of “positive chlorine”—in ethane-1,2-diol, obtaining the asymmetric compound **1**, as previously reported [31,41]. This synthetic strategy is based on the observation that the oxidative addition to Pt(II) complexes is generally assisted by the solvent coordinating opposite to the attacking “positive chlorine” [37,42,43]. In the present case, by using ethane-1,2-diol as solvent, this latter coordinates *trans* to chlorine [41]. The Pt(IV) complex **1** was then conjugated with **2** [34] forming the ester complex *cis*,*trans*,*cis*-[Pt(ethanedioato)Cl{2-(2-(4-(6,8-dichloro-3-(2-(dipropylamino)-2-oxoethyl)imidazo[1,2-*a*]pyridin-2-yl)phenoxy)acetate)-ethanolato}(1*R*,2*R*-DACH)] (**3**).

With reference to the coupling reaction, the best result was obtained using EDC and HOBt as coupling reagents in the presence of TEA in DMF; a similar synthetic approach was used for the conjugation of the glycolic monomer of PEG with PLGA [44].

Compound **3** was characterized by elemental analysis, ESI-MS, and NMR spectroscopy. In the ESI-MS spectrum a peak at *m*/*z* = 976.33, corresponding to [3 + Na]^+^, was evident (data not shown). The experimental isotopic pattern of this peak was in good agreement with the theoretical one.

**Figure 4:**
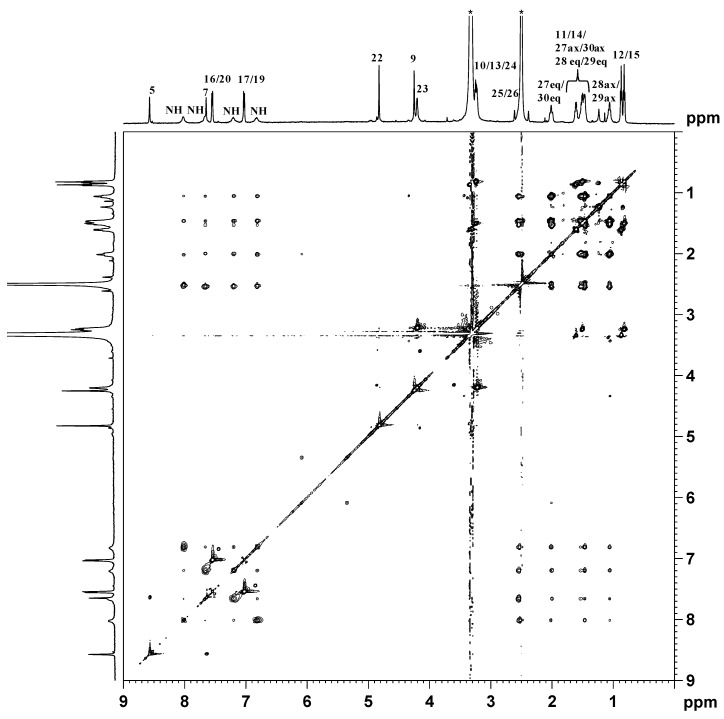
^1^H (**top** and **left**) and TOCSY 2D (**center**) NMR spectra (600 MHz, ^1^H) of **3** in DMSO-*d*_6_. The asterisks indicate residual solvent peaks.

The NMR characterization of the compound started from the assignment of methyl protons 12 and 15 (triplets falling at 0.82 and 0.87 ppm) of the dipropylacetamidic chains (see Figure 3 for numbering of protons), that show TOCSY cross-peaks (Figure 4) with methylenic protons 11 and 14 (overlapping multiplets at 1.51 and 1.61 ppm). These signals show TOCSY cross-peaks with two signals, overlapping with the signal of the solvent, assigned to methylenes 10 and 13 falling at 3.34 and 3.25 ppm. The two singlets falling at 4.25 and 4.83 ppm were assigned to the methylene groups 9 and 22, respectively.

**Figure 5:**
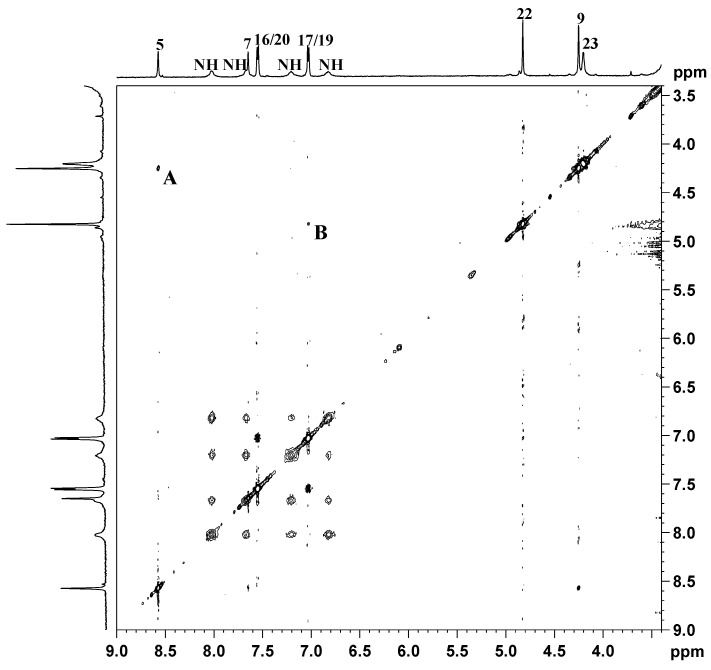
Portion of ^1^H (**top** and **left**) and NOESY 2D (**center**) NMR spectra (600 MHz, ^1^H) of **3** in DMSO-*d*_6_.

This assignment was confirmed by a NOESY 2D NMR experiment that shows a NOESY cross-peak between the methylene group 9 and the imidazopyridine proton 5 located at 8.57 ppm (cross-peak **A** in Figure 5), and an additional NOESY cross-peak of the methylene 22 with the protons 17 and 19 of the phenoxy ring located at 7.03 ppm (cross-peak **B** in Figure 5).

Proton 7 of the imidazopyridine ring was assigned to the singlet at δ = 7.65 ppm while the doublet located at 7.55 ppm, integrating for two protons, was assigned to protons 16 and 20 of the phenoxy ring (Figure 4, TOCSY cross-peak with protons 17 and 19). With reference to the hydroxyethanolato spacer, the multiplet at 4.20 ppm was assigned to the methylenic protons 23. This signal shows a TOCSY cross-peak (Figure 4) with the multiplet located at 3.23 ppm (overlapping with the signal of water) assigned to methylene 24. The 1*R*,2*R*-cychlohexanediamine is in a chair conformation and, owing to the axial asymmetry of the Pt complex, different signals are expected for both the axial and the equatorial protons. Therefore, the multiplet at 1.06 ppm was assigned to the axial protons 28 and 29, which showed TOCSY cross-peaks with the multiplet at 1.43–1.50 ppm (assigned to the axial protons 27 and 30 and to the equatorial protons 28 and 29 of 1*R*,2*R*-DACH), the multiplet at 2.01 ppm (attributed to equatorial protons 27 and 30), and two signals overlapping with the solvent (assigned to the methynic protons 25 and 26). All the chemical shifts found for the methylenic and methynic protons of DACH are in good agreement with those reported for similar asymmetric Pt(IV) octahedral complexes having a chlorido and glycolic moiety in the axial positions [41].

As far as the amine groups are concerned, it is possible to observe (Figure 4, top) the presence of four different signals for the NH_2_ protons of coordinated DACH located at 6.83, 7.21, 7.67 and 8.02 ppm (only two signals are observed in the case of complexes with equal axial ligands).

**Figure 6:**
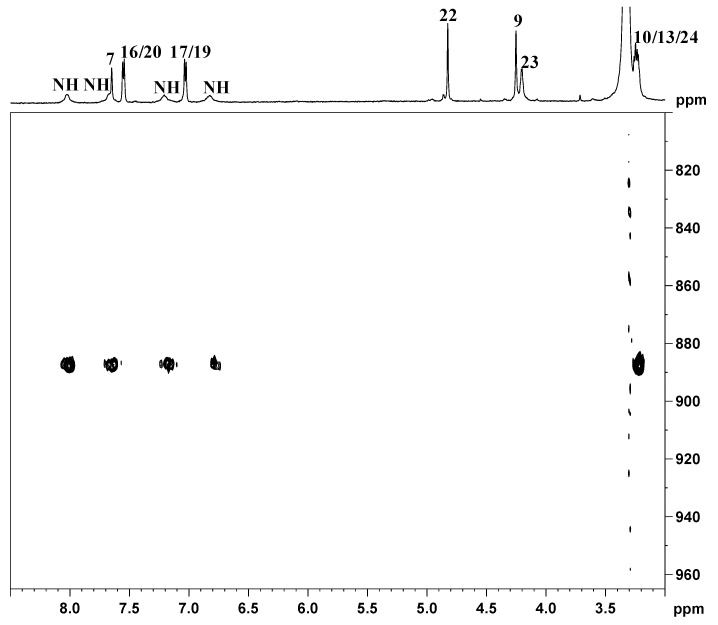
[^1^H-^195^Pt]-HSQC 2D NMR (300 MHz, ^1^H) of **3** in DMSO-*d*_6_.

**Figure 7:**
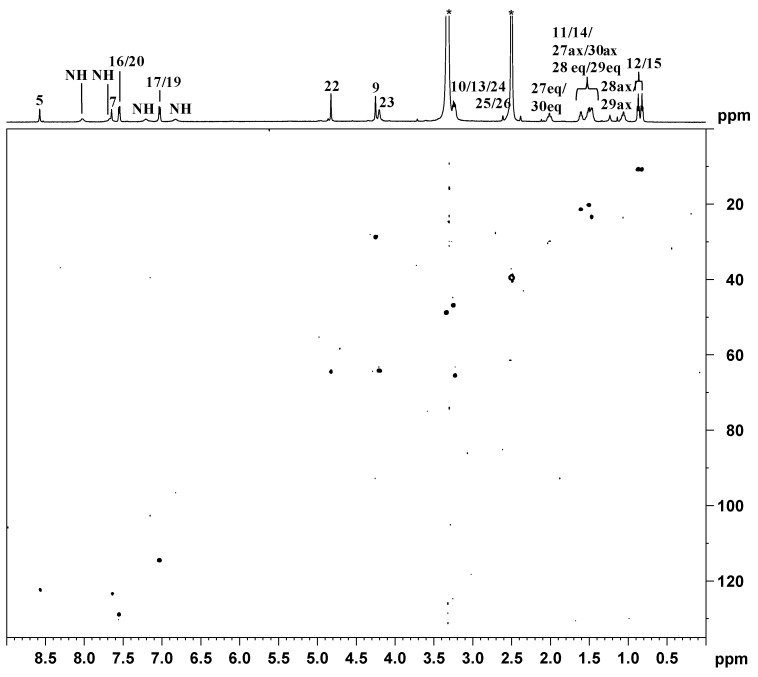
^1^H (**top**) and [^1^H-^13^C] HSQC 2D (**bottom**) NMR (600 MHz, ^1^H) spectra of **3** in DMSO-*d*_6_. The asterisks indicate residual solvent peaks.

The [^1^H-^195^Pt]-HSQC 2D NMR spectrum recorded in DMSO-d_6_ is reported in Figure 6. The spectrum shows four cross peaks falling at 6.83/887.2, 7.21/887.2, 7.67/887.2, and 8.02/887.2 ppm (^1^H/^195^Pt) due to the coupling of ^195^Pt with the four magnetically non-equivalent NH_2_ protons of DACH. In addition, a cross-peak located at 3.23/887.2 ppm is assigned to the coupling between ^195^Pt and the CH_2_ of the glycolic linker. The ^195^Pt chemical shift is in good agreement with those reported for similar asymmetric Pt(IV) octahedral complexes with a PtClN_2_O_3_ core [41]. The assignment of ^13^C signals has been accomplished by a [^1^H-^13^C]-HSQC 2D NMR spectrum (Figure 7 and Table 1). The chemical shifts found for 1*R*,2*R*-DACH and the glycolic linker are in good agreement with those reported for similar Pt(IV) complexes [41]. In particular, the methylene groups of DACH fall at 23.5 and 30.1 ppm, two values very similar to those reported for the product of esterification between BOC-l-alanine and *cis*,*trans*,*cis*-[Pt(cyclobutane-1,1′-dicarboxylate)Cl(2-hydroxyethanolato)(1*R*,2*R*-DACH)] (23.5, 30.1 and 30.7 ppm) [41]. The cross peak at 2.54/61.5 ppm (^1^H/^13^C) was assigned to the methynic group of DACH while the cross-peaks at 3.23/65.5 and 4.20/64.3 ppm (^1^H/^13^C) were assigned, respectively, to C24 and C23 of the glycolic moiety (66.0 and 64.5 ppm in the ester compound between BOC-L-alanine and *cis*,*trans*,*cis*-[Pt(cyclobutane-1,1′-dicarboxylate)Cl(2-hydroxyethanolato)(1*R*,2*R*-DACH)] mentioned above) [41]. The cross-peaks located at 122.3 and 123.5 ppm (^13^C) were assigned to C5 and C7 of the imidazopyridine ring, respectively. The cross peaks falling at 7.03/114.5 and 7.55/129.0 ppm (^1^H/^13^C) were assigned to carbon atoms 16/20 and 17/19 of the phenyl ring, respectively. The ^13^C chemical shift of methylene groups 9 and 22 fall at 28.7 and 64.4 ppm, respectively. The dipropylacetamidic chain gives two cross peaks at 0.82/10.9 and 0.87/10.9 ppm for the two methyl groups, two signals at 20.3 and 21.5 ppm belonging to methylene groups 11 and 14, and two signals at 46.9 and 48.9 ppm belonging to methylene groups 10 and 13.

### 2.2. Stability of **3**

**Figure 8:**
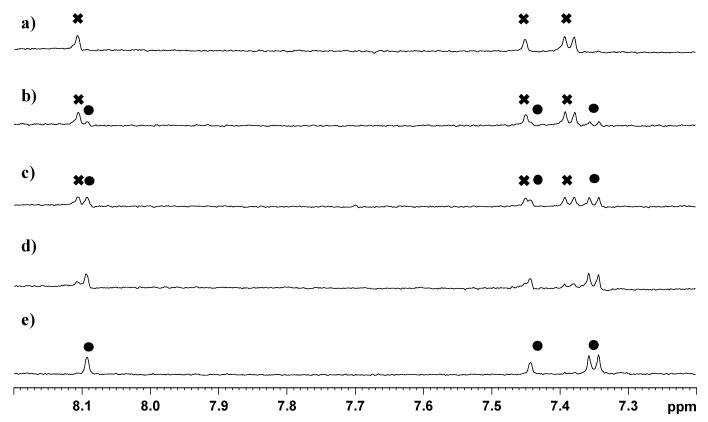
Portion of the ^1^H NMR (600 MHz, ^1^H) spectra of compound **3** dissolved in DMSO-d_6_/D_2_O (5:95 *v*/*v*) and incubated at pH 7.4 (2 mM phosphate buffer) and 37 °C. Spectra were recorded at zero time (**a**) and after 8 (**b**); 24 (**c**); 56 (**d**); and 108 h (**e**). 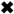 indicates peaks relevant to the portion of TSPO ligand in complex **3**. ● indicates peaks of free TSPO ligand (**2**). The amine protons were deuterated due to the fast H/D exchange.

The stability of **3** was investigated by ^1^H-NMR spectroscopy in DMSO-*d*_6_/D_2_O (5:95, *v*/*v*) at pH 7.4 (2 mM phosphate buffer) and 37 °C. A portion of the spectra, recorded at different times, is reported in Figure 8. The spectrum acquired soon after dissolution shows only the peaks of **3** (H5, 8.11 ppm; H7, 7.45 ppm; H16/20, 7.39 ppm; marked with 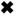 in Figure 8a). After 8 h of incubation we observed a decrease in intensity of the peaks of **3** and the concomitant increase of a new set of peaks, marked with ● in Figure 8b (H5, 8.09 ppm; H7, 7.44 ppm; H16/20 7.35 ppm), that was assigned to compound **2** by comparison with a spectrum of the TSPO ligand recorded in similar conditions. This new set of signals indicates that hydrolysis of the ester bond linking **2** to the ethanolato linker occurs in physiological-like conditions. The spectra recorded at 24, and 56 h showed a further decrease of the signals of **3**, which disappeared completely after 108 h. The half-life of the complex, as calculated from the NMR experiment, was determined to be approximately 24 h.

An HPLC analysis of the sample was performed after 108 h incubation at 37 °C. Two peaks were visible in the chromatogram (not shown) having retention times of 8.7 and 21.81 min, which are comparable to those found for the Pt(IV) precursor **1** and the free TSPO ligand **2**, respectively. Therefore, the HPLC investigation confirmed that **3** undergoes a slow hydrolysis of the ester bond in physiological-like conditions. No reduction of the Pt(IV) complex occurred under the conditions used in this experiment.

### 2.3. Biological Assays

The affinity towards TSPO of complex **3** and of the free ligand **2** was evaluated by testing their ability to prevent binding to the rat cerebral cortex of the selective TSPO ligand [^3^H]-PK 11195. The results, expressed as inhibitory concentration (IC_50_), are listed in Table 2. Compound **3** showed high affinity for the TSPO receptor (IC_50_ of 18.64 nM), although it was lower than that found for the free ligand **2** (IC_50_ of 2.12 nM). The free ligand **2**, in turn, was more affine than the reference ligand PK 11195 (IC_50_ of 4.27 nM). These results indicate that the choice of a terminal carboxylic residue on the free ligand **2** for conjugation to a Pt(IV) complex was correct and does not significantly alter the affinity of **2** for the TSPO.

The cytotoxic activity of complex **3** and of its precursors was assessed by the MTT in vitro assay performed in human MCF7 breast carcinoma, human U87 glioblastoma, and human LoVo colon adenocarcinoma cell lines. The cytotoxicity of the compounds, expressed as IC_50_ values after 72 h incubation (except in two cases where it is expressed as the % of cell viability at the maximum concentration tested of 100 μM) are reported in Table 3.

Compound **2** showed only a marginal cytotoxic effect at 100 μM concentration (the highest concentration used in the investigation) in the case of MCF7 and LoVo cells and a weak activity against U87 cell line (IC_50_ = 70.2 μM). Moreover, we could assess that compound **2** causes cell death through induction of apoptosis as evidenced by the formation of a sub-G0/G1 cell population (cell cycle analysis of LoVo cell line, see following discussion). These results are in line with previous results indicating that TSPO ligands, constructed on the imidazopyridinic scaffold, are endowed with good proapoptotic activity [30] although, in some cases, high concentrations are needed to achieve cytotoxic and proapoptotic effects, as also demonstrated for PK 11195 [45]. In particular, Kugler et al. [45] evidenced a peculiar property of TSPO ligands: being inert when no challenge is present but counteracting programmed cell death when lethal agents are present. They are therefore recognized to be potentially useful for the treatment of brain trauma and neurodegenerative brain diseases [45]. In the present case it is difficult to say if the low activity of compound **3** is due to the TSPO ligand **2** preventing cell death induction by oxaliplatin or to the intrinsic lower activity in cellular experiments of Pt(IV) species as compared to their Pt(II) counterparts. In this regard, further experiments will be carried out by giving the ligand **2** and the oxaliplatin derivative **1** simultaneously but as individual components.

In all three cell lines compound **3** had activity comparable to that of the Pt(IV) precursor complex **1** (IC_50_ ratios of 1.7, 1.8, and 0.92 for MCF7, U87, and LoVo cell lines, respectively). In the above mentioned cell lines both Pt(IV) compounds were less active than oxaliplatin. This latter result is not surprising since Pt(IV) compounds usually show higher IC_50_ values in in vitro investigation with respect to the related Pt(II) compounds since they require a preliminary intracellular reduction process before exerting their cytotoxic effect [46].

As already reported [8], oxaliplatin is a chemotherapeutic drug mainly used for the treatment of colorectal cancer, therefore, we extended our investigation only to LoVo cells. First we measured the intracellular accumulation of platinum by ICP-MS. LoVo cells were exposed to IC_50_ concentrations of compounds **3**, **1**, and oxaliplatin for a short (4 h) and a long (24 h) period. Interestingly, the results (Table 4) indicate that for 24 h incubation the uptake of compound **3** is greater than that of **1** and that the latter is greater than that of oxaliplatin (with a ratio of 2 between the uptake of **3** and that of oxaliplatin). Interestingly, for the shorter time (4 h) the Pt uptake follows the order **3** ˃ oxaliplatin ˃ **1** indicating that factors other than passive diffusion might play a role at short time exposure. Most likely, organic cation transporters (OCT) may facilitate the uptake of oxaliplatin [47] at short time exposure and then, after saturation, the passive diffusion of the most lipophilic compounds becomes prominent.

There does not appear to be a direct correlation between Pt uptake and cytotoxic effect (72 h) but, in our opinion, this is an intrinsic limitation of cellular testing where Pt(IV) compounds, which require reductive activation, generally result in less cytotoxicity than Pt(II) species and the directing role of TSPO ligands might remain hidden. In this case a more reliable answer can come from in vivo experiments which are being planned.

**Figure 9:**
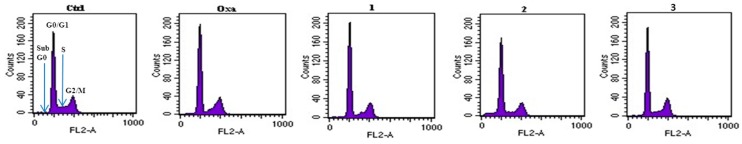
Flow cytometric analysis of LoVo cells stained with propidium iodide after 24 h of treatment with test compounds.

The capability of the new compounds **1** and **3** to perturb the cell cycle progression of LoVo cells at their IC_50_ concentrations was investigated by FCM analysis and compared to that of oxaliplatin and of the TSPO ligand **2** (Figure 9). After 24 h treatment with the test compounds, cells exhibited only a slight modification of the cell cycle distribution. As expected, oxaliplatin delayed the S-phase and started to accumulate cells in G2/M-phase and G0/G1 phase [48]. Compounds **3** and **1** induced no notable changes compared to oxaliplatin. The TSPO ligand **2** induced a sub-G0/G1 cell population after 24 h, consistent with the formation of apoptotic cells as previously demonstrated for this class of compounds [30].

Compound **3** did not induce apoptosis as the free TSPO ligand **2** did, however, this is because the concentration of compound **2** released after intracellular hydrolysis of compound **3** is not sufficient to exert apoptosis (note that compound **2** was administered at a much higher dose due to its lower cytotoxicity).

## 3. Experimental Section

### 3.1. Materials and Methods

Commercial reagent grade chemicals and solvents were used as received without further purification.

^1^H-NMR, COSY, TOCSY, NOESY, and [^1^H-^13^C]-HSQC 2D NMR spectra were recorded on a Bruker Avance III 600 MHz instrument (Bruker Italia S.r.l., Milano, Italy). Spectra of [^1^H-^195^Pt] HSQC 2D NMR were recorded on a Bruker Avance DPX 300 MHz instrument (Bruker Italia S.r.l., Milano, Italy). Chemical shifts of ^1^H and ^13^C were referenced using the internal residual peak of the solvent (DMSO-*d*_6_: 2.50 ppm for ^1^H and 39.51 ppm for ^13^C). ^195^Pt NMR spectra were referenced relative to K_2_PtCl_4_ (external standard placed at −1620 ppm with respect to Na_2_[PtCl_6_]) [49].

Electrospray Mass Spectrometry (ESI-MS) experiments were performed with a dual electrospray interface and a quadrupole time-of-flight mass spectrometer (Agilent 6530 Series Accurate-Mass Quadrupole Time-of-Flight (Q-TOF) LC/MS, Agilent Technologies Italia S.p.A., Cernusco sul Naviglio, Italy).

Elemental analyses were carried out with an Eurovector EA 3000 CHN instrument (Eurovector S.p.A., Milano, Italy).

Oxaliplatin was synthesized as previously reported [50].

*Cis*,*trans*,*cis*-[Pt(ethanedioato)Cl(2-hydroxyethanolato)(1*R*,2*R*-DACH)] (**1**) [31] and 2-(4-(6,8-dichloro-3-(2-(dipropylamino)-2-oxoethyl)imidazo[1,2-*a*]pyridin-2-yl)phenoxy)acetic acid (**2**) [34] were prepared according to already reported procedures.

### 3.2. Synthesis of cis,trans,cis-[Pt(ethanedioato)Cl{2-(2-(4-(6,8-dichloro-3-(2-(dipropylamino)-2-oxoethyl)imidazo[1,2-a]pyridin-2-yl)phenoxy)acetate)-ethanolato}(1R,2R-DACH)] (**3**)

A solution of **2** (37.6 mg, 0.079 mmol) and 1-Ethyl-3-(3-dimethylaminopropyl)carbodiimide (EDC, 18 μL, 0.102 mmol) in 2 mL of dry DMF was stirred at room temperature. After 5 min, 1-hydroxybenzotriazole hydrate (HOBt·H_2_O, 15.6 mg, 0.102 mmol) was added and the mixture was stirred at room temperature for 10 min. Then, a solution containing **1** (38.8 mg, 0.079 mmol) and triethylamine (TEA, 14 μL, 0.102 mmol), dissolved in 3 mL of dry DMF, was added dropwise and the reaction mixture was left stirring in the dark at room temperature. After 24 h, the solvent was removed by evaporation under reduced pressure. Compound **3** was purified by direct-phase chromatography using silica gel as stationary phase and a solution of 90:10 chloroform/methanol as eluent (Rf = 0.4). Yield: 21 mg (28%). *Anal.: calculated for* C_33_H_42_Cl_3_N_5_O_9_Pt·2H_2_O (**3**·2H_2_O): C, 40.03; H, 4.68; N, 7.07%. *Found*: C, 39.84; H, 4.60; N, 6.87%. ESI-MS: *calculated for* C_33_H_42_Cl_3_N_5_O_9_PtNa [1 + Na]^+^: 976.15. *Found: m*/*z* 976.33. ^1^H NMR (DMSO-*d*_6_): δ = 0.82 (t, 3H, *J* = 7.2 Hz, CH_3_, H_12_ or H_15_), 0.87 (t, 3H, *J* = 7.2 Hz, CH_3_, H_12_ or H_15_), 1.06 (m, 2H, CH_2(ax)_, H_28ax_/H_29ax_), 1.43–1.50 (m, 4H, CH_2(ax)_, CH_2(eq)_, H_27ax_/H_30ax,_ H_28eq_/H_29eq_), 1.51 (m, 4H, CH_2_, H_11_ or H_14_), 1.61 (m, 4 H, CH_2_, H_11_ or H_14_), 2.01 (m, 2H, CH_2(eq)_, H_27eq_/H_30eq_), 2.54 (overlapping with the signal of DMSO-d_6_, 2H, CH, H_25_/H_26_), 3.23 (m, 2H, CH_2_, H_24_), 3.25 (m, 2H, CH_2_, H_10_ or H_13_), 3.34 (overlapping with the signal of the residual water, 2H, CH_2_, H_10_ or H_13_), 4.20 (m, 2H, CH_2_, H_23_), 4.25 (s, 2H, CH_2_, H_9_), 4.83 (s, 2H, CH_2_, H_22_), 6.83 (b, 1H, NH_2_), 7.03 (d, *J* = 8.2 Hz, 2H, CH, H_17_/H_19_), 7.21 (b, 1H, NH_2_), 7.55 (d, *J* = 8.2 Hz, 2H, CH, H_16_/H_20_), 7.65 (s, 1H, CH, H_7_), 7.67 (b, 1H, NH_2_), 8.02 (b, 1H, NH_2_), 8.57 (s, 1H, CH, H_5_). ^13^C NMR (DMSO-*d*_6_): δ = 10.9 (C_12_/C_15_), 20.3 (C_11_ or C_14_), 21.5 (C_11_ or C_14_), 23.5 (CH_2_ DACH), 28.7 (C_9_), 30.1 (CH_2_ DACH), 46.9 (C_10_ or C_13_), 48.9 (C_10_ or C_13_), 61.5 (CH DACH), 64.3 (C_23_), 64.4 (C_22_), 65.5 (C_24_), 114.5 (C_17_/C_19_), 122.3 (C_5_), 123.5 (C_7_), 129 (C_16_/C_20_) ppm. See Figure 3 for numbering of protons.

### 3.3. Stability of Compound **3**

The stability of compound **3** in a physiological-like solution was investigated by NMR and HPLC.

Because of the poor water solubility of **3**, the complex was previously dissolved in DMSO-*d*_6_ and then diluted with deuterated aqueous phosphate buffered saline solution (2 mM, pH 7.4) obtaining a final concentration of 52 μM in DMSO-*d*_6_/D_2_O (5:95, *v*/*v*). The sample was incubated at 37 °C in the dark and ^1^H-NMR spectra recorded at different time intervals.

The NMR solution was further analyzed by HPLC analysis. Stationary phase: Waters Symmetry RP-C18 column, 5 μm, 4.6 × 250 mm, 100 Å. Mobile phase: phase A = water and phase B = Acetonitrile; isocratic elution 5% phase B for 5 min, linear gradient from 5% to 50% phase B in 10 min, isocratic elution 50% phase B for 7 min, linear gradient from 50% to 5% phase B in 2 min, isocratic elution 5% phase B for 6 min. Flow rate = 0.7 mL·min^−1^. UV-visible detector set at 220 nm.

### 3.4. Biological Assays

#### 3.4.1. Cell Lines

Human MCF7 breast carcinoma, human U87 glioblastoma, human LoVo colon adenocarcinoma cell lines and C6 rat glioma cells were used. As recommended for these cell lines, the base medium used (EuroClone, Pero (MI), Italy) was RPMI for MCF7 cells, DMEM for U87 and C6 cells, and F-12/HAM for LoVo cells, enriched with fetal bovine serum (10%), penicillin (100 U/mL)/streptomycin (100 μg/mL) (1%) and glutamine 200 mM (1%) (EuroClone, Pero (MI), Italy). Cells were incubated at 37 °C under an atmosphere containing 5% CO_2_.

#### 3.4.2. Membrane Preparation

Membranes from C6 rat glioma cells were prepared as described by Piccinonna et al. [51] with minor modifications. In brief, cells scraped in PBS and harvested, were then homogenized with a Brinkman Polytron (Kinematica AG, Münstertäler, Germany). After centrifugation at 37,000× *g* for 30 min at 4 °C the resulting pellet was resuspended in ice-cold 10 mM PBS and stored at −80 °C until use.

#### 3.4.3. Receptor Binding Assays

The ability of complex **3** and the precursor TSPO ligand **2** to bind with high affinity to TSPO was assessed by in vitro receptor binding assays performed as described in our previous works [49,52]. In brief, a suspension of C6 rat glioma cells membranes (100 μg) in PBS was treated with 0.7 nM [^3^H]-PK11195 (a selective ligand for TSPO) and compound **2** or **3** at different concentrations. After an incubation time of 90 min at 25 °C, the samples were rapidly filtered through Whatman GF/C glass microfiber filters and the filters washed with 3 × 1 mL of ice-cold PBS. For the determination of the nonspecific binding the compound PK 11195 (10 μM) was used. A specific binding equal to 90% was determined under these experimental conditions.

#### 3.4.4. Cell Proliferation Assay

Determination of cell growth inhibition was performed on MCF7, U87 and LoVo cell lines, using the 3-[4,5-dimethylthiazol-2-yl]-2,5-diphenyltetrazoliumbromide (MTT) assay. The determination of the % of cell viability after treatment with the test compounds, was performed as described by Denora et al. [34]. Briefly, cells were dispensed on 96 microtiter plates at a density of 5000 cells/well and, after overnight incubation, were exposed for 72 h to solutions of the compounds with concentration in the range 0.01–100 µM. After addition of MTT (10 μL of 0.5% *w*/*v* in PBS) and incubation for an additional 3 h at 37 °C, the cells were lysed with 100 μL of DMSO/EtOH 1:1 (*v*/*v*) solution, dispensed in each well. A PerkinElmer 2030 multilabel reader Victor TM X3 (manufactured for WALLAC Oy, Turku, Finland by PerkinElmer Singapore was used for the absorbance determination at 570 nm.

#### 3.4.5. Cell Cycle Analysis

Cells were dispensed in 60 mm tissue culture dishes at a density of 500,000 cells/dish and after 24 h were treated with compounds **1**, **3**, or oxaliplatin at their IC_50_ concentrations or with compound **2** at 100 µM for an additional 24 h. Then, the experimental procedure described in our previous work was performed [53].

#### 3.4.6. Cellular Uptake

A Varian 820-MS ICP mass spectrometer (Varian Italia, Cernusco sul Naviglio (MI), Italy) was used to perform the inductively coupled plasma mass spectrometry (ICP-MS) analyses for the determination of the platinum cellular uptake in LoVo colon cancer cells. Cells were seeded in 60 mm tissue culture dishes at a density of 500,000 cells/dish and incubated at 37 °C in a humidified atmosphere with 5% CO_2_. After 1 day, the culture medium was replaced with 3 mL of medium containing complex **3**, the precursor **1**, or oxaliplatin at their IC_50_ concentrations, and incubated for 4 and 24 h. After the incubation period, the cell monolayer was washed twice with ice-cold PBS and then digested with 2 mL of an HNO_3_(67%)/H_2_O_2_(30%), 1:1 (*v/v*), solution for 4 h at 60 °C in a stove. The platinum content was determined by ICP-MS.

## 4. Conclusions

The first Pt(IV) complex containing a TSPO ligand in the axial position has been synthesized, fully characterized, and its cytotoxic activity tested in vitro. The TSPO ligand in the axial position of the conjugate **3** maintains the high affinity for the TSPO receptor in C6 rat glioma cells at nanomolar level. Therefore, the new compound appears to be very promising in a receptor-mediated drug targeting context towards TSPO-overexpressing tumors, in particular colorectal cancer.

Indeed, the best cytotoxic effect of **3** was observed in LoVo colon cancer cells. However, the cytotoxicity of **3** was 5-fold lower than that of oxaliplatin, which is a good result considering the different oxidation state of the metal in **3** and in oxaliplatin.

Remarkably, compound **3**, which in MCF7 and U87 cell lines was less active than **1**, proved to be more active than **1** in LoVo cells. Since TSPO is overexpressed in colorectal cancer [19], an additional increase of potency of **3** with respect to **1** could, in principle, derive from the presence of a TSPO ligand in axial position.

We expect a clearer answer about the potential of these combination compounds from in vivo testing, where the required reductive activation of the Pt(IV) precursor, and the hydrolysis necessary for the release of the TSPO ligand might not represent a handicap as it does for in vitro cellular testing.

## Figures and Tables

**Table 1:** Summary of ^13^C chemical shifts of **3** in DMSO-*d*_6_.

C	δ ^13^C (ppm)
5	122.3
7	123.5
9	28.7
10 or 13	46.9
10 or 13	48.9
11 or 14	20.3
11 or 14	21.5
12/15	10.9
16/20	129
17/19	114.5
22	64.4
23	64.3
24	65.5
CH_2_ DACH	23.5
CH_2_ DACH	30.1
CH DACH	61.5

**Table 2:** Affinities of compounds **2** and **3** for TSPO from rat cerebral cortex.

Compound	IC_50_ (nM) ^a^
	TSPO ^b^
**2**	2.12 ± 0.10
**3**	18.64 ± 0.84
PK 11195	4.27 ± 0.22

^a^ Data are means of three separate experiments performed in duplicate which differed by less than 10%; ^b^ PK 11195, a selective ligand for TSPO 18-kDa, was used for comparison.

**Table 3:** Concentration inducing 50% cell survival inhibition (IC_50_) (except in two cases where is given the % of cell viability at the maximum concentration of 100 μM) after 72 h treatment.

Cell Lines	IC_50_ (µM) or % Cell Viability at 100 µM			
	1	2	3	Oxaliplatin
MCF7	8.2 ± 0.4	53% ± 1%	14.1 ± 0.1	5. 4 ± 0.4
U87	9.1 ± 0.4	70.2 ± 0.3 (µM)	16.1 ± 0.3	3.1 ± 0.2
LoVo	2.5 ± 0.5	65% ± 4%	2.3 ± 0.1	0.46 ± 0.01

**Table 4:** Uptake by LoVo colon cancer cells of oxaliplatin and compounds **1** and **3**.

Treatment Time	Uptake by LoVo Cells (ppb of Pt)		
	Oxaliplatin	1	3
after 4 h treatment	0.24 ± 0.04	0.18 ± 0.01	0.33 ± 0.02
after 24 h treatment	0.38 ± 0.01	0.59 ± 0.03	0.77 ± 0.02

## Abbreviations

COSY	correlation spectroscopy
DACH	diaminocyclohexane
DMF	dimethylformamide
DMSO	dimethylsulfoxide
EDC	1-Ethyl-3-(3-dimethylaminopropyl)carbodiimide
ESI-MS	electrospray mass spectrometry
HOBt	1-hydroxybenzotriazole hydrate
HSQC	heteronuclear single quantum coherence spectroscopy
ICP-MS	inductively coupled plasma mass spectrometry
NOESY	nuclear overhauser enhanced spectroscopy
OCT	organic cation transporters
PEG	polyethylene glycol
PLGA	poly(lactic-co-glycolic acid)
PET	positron emission tomography
TEA	triethylamine
TOCSY	total correlation spectroscopy
TSPO	18-kDa mitochondrial translocator protein
